# A Call for Rigorous Clinical Trial: Effect of Intratympanic Dexamethasone on Bell’s Palsy

**DOI:** 10.22038/ijorl.2025.85294.3866

**Published:** 2026

**Authors:** Srilakshmi R Rao, Chethana Ramesh, Pranaya Deepika

**Affiliations:** 1 *Department of ENT, Bharati Vidyapeeth Medical College, Pune, India, 411043*

Greetings! We are hereby writing regarding the article “Effect of Intratympanic Dexamethasone on Bell’s palsy: A Clinical Trial” by Ali Bagherihagh et al. published in your esteemed journal in the November 2024, Volume 36, issue (6). 

We are hereby writing to bring to your attention some concerns regarding the article. We understand that scientific publishing is indeed a challenge and our concerns will be considered in a constructive way.

Firstly, though the study has a clearly defined research question, the sampling technique did not ensure comparable group sizes impacting the power of the study and also generalisabilty as intervention group had 54 patients and the control group 123 patients. Secondly, the exact timing of patient presenting within the first 14 days of onset of symptoms remained unclear. Given the strong association between early initiation of treatment and improved recovery (1), acccuarate documentation of presentation time is of utmost importance.

While we do understand that the study design is ‘Clinical trial’ and not ‘Randomised control Trial’ potential confounding factors like age of the patient, initial House Brackmann grading before progression to total paralysis (2), implementation and compliance to facial physiotherapy (3), may have influenced the results of the study. These factors were not considered in the study design. The study raises concern about the methodological precision like inappropriate sampling technique, ignoring a steroid tapering regimen adjusted as per patient’s clinical response based on House Brackmann staging, study has also not addressed potential influence of attrition bias. 

The study mentions ear perforation as a major complication in the intervention group however lacked quantitative data pertaining to this in the study population.

Overall, we believe that the study provides valuable preliminary information about the effect of Intratympanic dexamethasone in the management of Bell’s palsy nevertheless a well-designed clinical trial with exhaustive methodology including matching participants across groups, standardized patient management protocols, rigororus data collection would have further strenghthened the internal validity of the study.

We hope that these points stimulate discussion, help in future research and advancement of knowledge. We would be grateful if you could review the matter and if you find this appropriate consider addressing it in a future issue. Thank you for your time. Your response is awaited.

## Response from Corresponding author:

We sincerely appreciate the interest of the readers in our article titled “Effect of Intratympanic Dexamethasone on Bell’s Palsy: A Clinical Trial” published in the November 2024 issue of your esteemed journal. We welcome the opportunity to respond to the points raised and provide clarifications where necessary. Below we address each of the concerns in order:

1. Concerning the imbalance in sample size between intervention and control groups:

We thank the authors for pointing this out. The allocation of patients into the two groups was performed in a semi-randomized manner using blocks of 4 and 6 patients, based on availability and practical constraints such as drug accessibility in our outpatient clinic. While the final numbers were unequal (54 vs. 123), statistical analysis demonstrated that the groups were homogeneous in terms of age, gender, and initial severity of facial palsy (all patients had House-Brackmann grade 6). Therefore, a fair degree of comparability was maintained.

2. Regarding the timing of patient presentation within 14 days:

As reported in the 'Materials and Methods' section, the average interval from symptom onset to initiation of treatment was 3.39 days in the intervention group and 3.25 days in the control group, with no statistically significant difference (P=0.585). Hence, the documentation of symptom onset and early treatment initiation was both accurate and comparable between groups.

3. Concerning potential confounders such as age, initial House-Brackmann grading, and adherence to physiotherapy:

All patients were enrolled at the same stage of disease severity (House-Brackmann grade 6), ensuring uniformity at baseline. Age and gender were statistically comparable between the two groups (Table 2, P > 0.7). While we acknowledge that adherence to facial physiotherapy was not explicitly measured, all patients were managed in the same clinical setting and provided similar instructions. Nevertheless, we accept that physiotherapy compliance was not controlled and acknowledge this as a limitation for future studies.

4. Concerning methodological aspects including steroid tapering, attrition bias, and complication data:

All patients received a fixed dose of oral prednisolone at 1 mg/kg/day for 10 days. This dosing schedule was based on a protocol designed by our research team and referenced from similar studies. According to current endocrinological understanding, steroid tapering is not required for corticosteroid use under 14 days, even at high doses, since hypothalamic-pituitary-adrenal (HPA) axis suppression does not occur in this duration.

Regarding attrition bias, we reported in the Results section that 8 patients were excluded due to loss to follow-up, with a final analyzed sample of 177. Although no formal attrition analysis was conducted, the low dropout rate suggests limited impact on the study outcomes. We recognize this as an area for improved reporting in future work.

As for adverse events, we explicitly stated that no major complications such as tympanic membrane perforation occurred. Minor adverse effects, including transient otalgia and mild dizziness, were reported in 24% and 24.9% of patients in the intervention group, respectively. We agree that reporting the percentage of patients without complications would provide additional clarity, and we will address this in future reporting.

5. On the general comment regarding internal validity and study design:

We appreciate this constructive observation. As noted in the Discussion section of our paper, we openly acknowledged the limitations in randomization and sample size and encouraged future trials with more rigorous design to validate our findings. 

We believe this study offers valuable preliminary insights into intratympanic dexamethasone as an adjunct therapy in Bell’s palsy, and serves as a basis for further research. We thank the readers again for their critical engagement with our work and hope these clarifications contribute positively to the ongoing academic discourse.
